# Synergic effects of some plant-derived essential oils and Iranian isolates of entomopathogenic fungus *Metarhizium anisopliae* Sorokin to control *Acanthoscelides obtectus* (Say) (Coleoptera: Chrysomelidae)

**DOI:** 10.3389/fpls.2022.1075761

**Published:** 2022-12-09

**Authors:** Fatemeh Lak, Nooshin Zandi-Sohani, Mohammad Hamed Ghodoum Parizipour, Asgar Ebadollahi

**Affiliations:** ^1^ Department of Plant Protection, Faculty of Agriculture, Agricultural Sciences and Natural Resources University of Khuzestan, Mollasani, Iran; ^2^ Department of Plant Sciences, Moghan College of Agriculture and Natural Resources, University of Mohaghegh Ardabili, Ardabil, Iran

**Keywords:** biological control agent, compatibility, biorational insecticide, bean weevil, essential oil

## Abstract

**Introduction:**

The bean weevil, *Acanthoscelides obtectus*, is one of the most important pests of the common bean, *Phaseolus vulgaris*. The pest attacks *P. vulgaris* seeds while they are still in the field. However, the damage continues during storage, where it causes the most significant losses.

**Methods:**

The present study was conducted to evaluate the insecticidal activity, and synergic effects of three essential oils (EOs) extracted from fennel (*Foeniculum vulgare*), tarragon (*Artemisia dracunculus*), and lavender (*Lavandula angustifolia*), and three isolates from an entomopathogenic fungus (EPF), *Metarhizium anisopliae*, including IRAN2273C, IRAN2252C, and IRAN1018C against the adults of *A. obtectus*. The effects of EOs were also evaluated on mycelial growth and conidiation of the fungal isolates.

**Results and Discussion:**

The results showed that all the EOs and the EPF exhibited insecticidal activity against *A. obtectus*. According to calculated LC_50_, *L. angustifolia* (1.2526 µl/l) and *F. vulgare* (0.9247 µl/l) EOs caused significantly higher mortality than *A. dracunculus* (3.1980 µl/l) against *A. obtectus.* The results of the pathogenicity of M. *anisopliae* isolates revealed that all isolates had insecticidal activity against *A. obtectus*. The cumulative mortality of insects varied from 59.12% in IRAN1018C to 80.86% in IRAN2273C. According to the compatibility test results, all EOs were compatible with fungal isolates except for *A. dracunculus*, which was toxic to the IRAN2252C isolate and showed incompatibility. The mortality of *A. obtectus* adults differed significantly among combined treatments of EOs and *M. anisopliae* isolates. According to the calculated synergic ratio, combinations of essential oils and fungal isolates had additive or synergistic effects on the mortality of *A. obtectus*. Based on the present findings, *A. obtectus* adults were susceptible to fennel, and lavender EOs, and their mortality was amplified when the EOs were combined with *M. anisopliae* isolates. These results can be helpful for the integrated management of *A. obtectus* during storage.

## 1. Introduction

A considerable proportion of stored agricultural products is destroyed annually due to quantitative and qualitative damage caused by insect pests (Nayak and Daglish, 2018). In addition to heavy losses in yield production, the pests endanger the health of consumers, including humans, livestock, and poultry (Tripathi, 2018).

Legumes are a source of carbohydrates, calcium, iron, and protein and are considered the second-largest source of human food after cereals (Tharanathan and Mahadevamma, 2003). The bean weevil, *Acanthoscelides obtectus* (Say) (Coleoptera: Chrysomelidae: Bruchinae), is a severe post-harvest and field insect pest of common beans (*Phaseolus vulgaris* L.). It is originated from the Neotropical region and is now a cosmopolitan pest of stored legumes (Ghahari and Borowiec, 2017). In total, 117 species from 14 genera of the subfamily Bruchinae are listed as the fauna of Iran (Ghahari and Borowiec, 2017). *Acanthocelides obtectus* is the only species of the genus *Acanthoscelides* spp. reported from Iran (Ghahari and Borowiec, 2017). The pest may infest growing pods by chewing and laying their eggs as clusters into pod cavities. The newly hatched larvae penetrate the beans after wandering around them for a while (Parsons and Credland, 2003). Adults mate after 24 h of their emergence and begin oviposition the next day. The majority of eggs are released freely among the seeds and are never stuck to them (Parsons and Credland, 2003). In Iran, 10 to 20% of storage products are destroyed annually by pests. however, in some rural areas, due to the usage of traditional warehouses, the amount of damage reaches up to 80% (Schalk and Rassoulian, 1973).

Synthetic fumigants such as methyl bromide and phosphine are mainly used to control storage pests. However, their use is currently limited due to their extreme toxicity to human and environmental contamination (Nyamador et al., 2010; Napoleão et al., 2015). Various methods have been introduced to replace chemical insecticides for controlling storage pests, including biocontrol, storage climate control, and the use of ionizing radiation (Daglish et al., 2018). Entomopathogenic fungi (EPF) are considered a promising tool for pest biocontrol globally (Skinner et al., 2014). According to their eco-friendly aspects and insecticidal effectiveness, plant-derived essential oils (EOs) have also been assayed as promising alternatives to commercial pesticides (Isman and Grieneisen, 2014; Ebadollahi and Jalali Sendi, 2015; Ebadollahi et al., 2020). *Metarhiazium anisopliae* is an important EPF that causes green muscardine disease in insects (Reddy et al., 2014). It has been highly recommended that EPF are applied in combination with other control means, such as plant-derived essential oils (EOs), which increases insect control efficiency (Borgio et al., 2008; Mohamed, 2009; Kovendan et al., 2012; Murugan et al., 2014; Batta and Kavallieratos, 2018). However, some incompatible relationships have been found between EPF and EOs, which restrict the simultaneous application of these control tools (Akbar et al., 2005; Mohamed, 2009; Eckard et al., 2017). Therefore, EPF-EOs interactions needed to be investigated before their application against insect pests.

Since there was no information on interactions between *Metarhizium anisopliae* (Metschn.) Sorokin and EOs against *A. obtectus*, this study was conducted to investigate the insecticidal efficacy of this Iranian isolates of entomopathogenic fungus including IRAN2273C, IRAN1018C, and IRAN2252C and EOs of lavender (*Lavandula angustifolia* Mill.), fennel (*Foeniculum vulgare* Mill.) and tarragon (*Artemisia dracunculus* L.) against the insect species.

## 2 Materials and methods

### 2.1 Insect rearing

The individuals of *A. obtectus* were collected from the pest-infected cowpea in a local shop in Azna city, Lorestan province, western Iran. One-liter cylindrical containers were used to rear the insects. Uninfected cowpeas were stored at -10°C for 72 h to eliminate possible pest infestation. Then 200 g of cowpea seeds were poured into each container, and 100 male and female insects were randomly transferred into them. The incubation conditions included a constant temperature of 28 ± 2°C, relative humidity of 60 ± 5%, and dark condition.

### 2.2 Essential oils

The EOs of lavender (*L. angustifolia*) and fennel (*F. vulgare*) were supplied by Johareh Ta’m Company (Mashhad, Iran), and the EO of tarragon (*A. dracunculus*) was supplied by Dorrin Golab Agro-Industry Company (Kashan, Iran). The EOs were stored at 4°C until the beginning of the experiments.

### 2.3 Fungi

Three fungal isolates of *M. anisopliae*, including IRAN2273C, IRAN1018C and, IRAN2252C, were obtained from the Institute of the Iranian Plant Protection Researches (Tehran, Iran). The fungi were sub-cultured on Potato Dextrose Agar (PDA) in 8-cm-diameter plates and incubated in darkness at 28°C for four weeks. The single spore method (Zhang et al., 2013) produced purified cultures for each fungal isolate. The viability of conidia was examined before the bioassay through a conidial germination test on a PDA medium after 24 h incubation. To make conidial suspensions, 12 mL of distilled deionized water (ddH_2_O) and Tween-80 (0.01%) solution was mixed with the 15-day-old PDA culture, and conidia in the mixture were harvested using a sterile glass rod. They were then filtered using cheesecloth (4 layers). A hemocytometer (HGB, Germany) was used to calculate the conidial concentration with three replications. To conduct experiments three conidial concentrations including 1.7×10^5^, 2.3×10^5^ and 7.9×10^5^ conidia/ml were prepared for IRAN2252C, IRAN2273C, and IRAN1018C, respectively.

### 2.4 Fumigant toxicity of EOs against adults of *A. obtectus*

Appropriate concentrations of EOs determined based on preliminary tests. 0.1, 0.2, 0.42, 0.87, and 1.8 µl/l air for fennel, 0.001, 0.003, 0.012, 0.042, and 0.15 µl/l air for lavender, and 0.1, 0.18, 0.34, 0.64, and 1.2 μl/l air for tarragon were prepared for concentration-mortality response tests. Filter papers with a diameter of 2 cm were attached to the inner surface of the vial caps with a volume of 50 ml. Desired concentrations of EOs were poured on each paper using a micropipette. Each concentration was replicated four times, and pure acetone (Merck, Germany) was used as a control. Twenty adult insects were placed in each vial, and covered using a net. Then the cap of the vials was screwed tightly and samples were kept at 28 ± 2°C under a relative humidity of 60 ± 5% and a photoperiod of 16:8 h (L: D). After 24 h, the number of dead insects was recorded.

### 2.5 Pathogenicity of fungi on adults of *A. obtectus*

Cherry et al. (2005) method was used to estimate the toxicity of fungal isolates. After preparing the conidial concentrations containing 0.01% Tween-80, ten female insects were immersed in conidial suspension for four seconds. Control samples were prepared by immersing insects in distilled water containing 0.01% Tween-80. The treated insects were transferred to sterile Petri dishes containing filter paper to dry their body surface. The insects were then transferred to 50 ml tubes containing 5 g of cowpea and kept at 25 ± 1°C. The conidia viability was tested before their application against the insect. To this end, one ml of each conidial suspension was fully spread onto the PDA culture media. The culture media was kept in darkness at 28 ± 1°C for 24 h. Conidia were randomly selected, and the number of germinated conidia was determined using a light microscope (Panahi et al., 2014). Experiments were replicated three times, and the insect mortality was recorded daily for seven days.

### 2.6 Effect of EOs on fungal growth and reproduction

#### 2.6.1 Effect on mycelial growth

The LC_50_ concentrations of EOs calculated from fumigant assays were added to fungal cultures by pouring on 8-cm-diameter filter paper embedded in the lid of Petri dishes. Pure acetone was used as a control. In order to prevent possible contamination or evaporation of EOs, Petri dishes were sealed with Parafilm. Then, they were incubated at 25 ± 1°C, 60 ± 5% RH, and in dark condition for 15 days. After that, the mycelial growth of the fungi in Petri dishes was measured using a ruler in two diameters perpendicular to each other. All experiments were replicated three times, and the percentage of inhibitory growth of the fungus was calculated using the formula below:


$$I=\frac{C-T}{C}\times 100$$

which *I* is the percentage of growth inhibition of treated samples (*T*) against control (*C*), and *C* and *T* are the hyphal extension of the colony (mm) in the control and plates treated with each EO, respectively. (Farzaneh et al., 2015).

#### 2.6.2 Effect on conidiation

In order to count the conidia produced in each treatment, a circle with a diameter of 10 mm was randomly cut from each Petri dish of the above experiments, using a sterilized metal loop 15 days post- incubation. Then, the samples were transferred into test tubes, and 10 ml of sterile distilled water containing 0.01% Tween-80 was added to the tubes. In order to separate the conidia from mycelia, the tubes were individually vortexed for 5 min at room temperature. The concentration of suspension was also determined, as described above.

#### 2.6.3 Compatibility calculation

To calculate the *in vitro* compatibility of EOs with EPF, the formula proposed by Neves et al. (2001) was used for toxicity classification. In this model, *VG* and *SP* are the percentages of mycelial growth and conidiation compared to the control, respectively. Then, the degree of compatibility of EOs was determined according to the *T* value calculated ((0 to 30 = very toxic; 31 to 45 = toxic; 46 to 60 = moderately toxic; > 60 = compatible)


$$T=\frac{20\left(VG\right)+80\left(SP\right)}{100}$$

#### 2.7 Combined effects of EPF and EOs

Combined effects of EOs and *M. anisopliae* isolates were evaluated using LC_25_ and LC_50_ of EOs and concentrations of 1.7×10^5^, 2.3×10^5^, and 7.9×10^5^ conidia/ml for IRAN2252C, IRAN2273C, and IRAN1018C isolates against *A. obtectus*, respectively. For this purpose, insects were immersed in conidial suspension and then transferred into glass containers containing five g of cowpea. The desired concentrations of plant EOs were poured on filter paper embedded in the lid of glass containers, and the lids were screwed tightly. To prevent the escape of EO vapor, the lids were covered with Parafilm. The experiment was carried out in five replications, and the mortality of insects was recorded after 24 h.

### 2.8 Data analysis

The mortality rates were corrected by the Abbott formula (Abbott, 1925). Analysis of variance and comparison of means was performed in a completely randomized design using Duncan’s multiple range tests. The values ​​of lethal and sub-lethal concentrations (LC_25_ and LC_50_) were calculated based on Probit analysis using SAS software (version 9.1 (SAS Institute Inc. Cary, NC). To determine the type of EO-fungus interaction, the synergistic ratio was calculated for each of the EOs and EPF according to the following formula:


$$SR=\frac{EX}{Ob}=\frac{A+B}{\left(A+B\right)}$$

where *A* is the mean mortality percentages of sublethal EO concentrations (LC_25_and LC_50_), *B* is the mortality of above-mentioned concentration of *M. anisopliae* isolates, and *A* + *B* and (*A* + *B*) are the expected and observed mortality rates, respectively. *SR* values less than 0.7, 0.7-1.8, and more than 1.8 indicate synergistic, cumulative, and antagonistic phenomena, respectively (Ebadollahi et al., 2017).

## 3 Results

### 3.1 Fumigant toxicity of EOs against *A. obtectus*

The results of fumigant toxicity tests of EOs extracted from lavender (*L. angustifolia*), fennel (*F. vulgare*), and tarragon (*A. dracunculus*) against *A. obtectus* adults are shown in **Table 1**. According to calculated LC_50_ and the 95% confidence limits, *L. angustifolia* (1.2526 µl/l) and *F. vulgare* (0.9247 µl/l) EOs caused significantly higher mortality than *A. dracunculus* (3.1980 µl/l) against *A. obtectus.*

**Table 1:** Fumigant toxicity of essential oils of from *L. angustifolia*, *F. vulgare* and *A. dracunculus* against *A. obtectus* adults.

Essential oils	LC_25_(µL L^-1^)	LC_50_(µL L^-1^)	Slope ± SE	Degree of freedom	Chi Square (χ^2^)
*L. angustifolia*	0.0507 (0.0242-0.1601)	1.2526 (0.3134-32.8033)	0.48 ± 0.10	3	1.06
*F. vulgare*	0.1566 (0.0778-0.2365)	0.9247 (0.6436-1.6007)	0.87 ± 0.15	3	5.99
*A. dracunculus*	0.6408 (0.4519-1.0697)	3.1980 (1.6601-13.8148)	0.96 ± 0.20	3	0.61

### 3.2 Pathogenicity of fungi on adults of *A. obtectus*

According to **Table 2**, the calculated LT_50_ values were 2.40, 3.41, and 2.72 days for IRAN2273C, IRAN1018C, and IRAN2252C isolates, respectively. However, LT50 values did not indicate a significant difference between isolates of *M. anisopliae* due to overlapping their confidence limits. The insect mortality ranged from 59.12% in IRAN1018C treatment to 80.86% for IRAN2273C (**Table 2**). The viabilities of IRAN2273C, IRAN1018C, and IRAN2252C isolates were determined as 97, 99, and 96%, respectively.

**Table 2:** Cumulative mortality and LT_50_ values calculated for entomopathogenic fungi against *A. obtectus* adults exposed at the concentrations used in the experiments (1.7×10^5^, 2.3×10^5^ and 7.9×10^5^ conidia ml^-1^ for IRAN2252C, IRAN2273C, IRAN1018C isolates, respectively).

Fungal isolate	Mortality (% ± SE)	LT_50_ (d) (95% FL)	Slope ± SE
IRAN2273C	80.86 ± 9.1	2.40 (1.98-3.12)	3.324 ± 0.362
IRAN1018C	59.12 ± 6.8	3.41 (2.51-4.2)	4.003 ± 0.456
IRAN2252C	73.57 ± 8.2	2.72 (2.3-3.21)	3.361 ± 0.393

SE, standard error; d, day; FL, fiducial limit.

### 3.3 Effect of EOs on fungal growth and reproduction

The compatibility tests of three isolates of *M. anisopliae* with *A. dracunculus, L. angustifolia*, and *F. vulgare* EOs showed that all EOs inhibited conidiation and mycelial growth of the fungi (**Table 3**). Among EOs, *A. dracunculus* had the highest inhibition effect on the conidiation (41.53%) and mycelial growth (30.17%) of IRAN2252C isolate. *Foeniculum vulgare* showed the most minor adverse effects on mycelial growth of the IRAN2273C; however, the least negative effect on conidiation was observed in *A. dracunculus* when applied against IRAN1018C isolate. According to the compatibility test results, all the EOs were compatible with the fungal isolates except for *A. dracunculus* EO, which was toxic to the IRAN2252C isolate and showed incompatibility (**Table 3**).

**Table 3:** Classification of *L. angustifolia*, *F. vulgare*, and *A. dracunculus* essential oils based on T values on IRAN2273C, IRAN1018C, and IRAN2252C isolates of *M. anisopliae*.

Essential oil	Fungal isolate	MGI (%±SE)	CI (%±SE)	T value	Compatibility index
*L. angustifolia*	IRAN2273C	12.42±1.7^b^	17.18±1.4^b^	79.53	C
	IRAN1018C	11.67±1.8^b^	22.17±1.6^a^	71.19	C
	IRAN2252C	22.59±2.1^a^	20.9±2.3^a^	71.68	C
*F. vulgare*	IRAN2273C	8.4±1.1.3^c^	12.82±1.3^b^	88.05	C
	IRAN1018C	18.13±1.9^b^	23.81±3.3^a^	77.31	C
	IRAN2252C	27.33±2.9^a^	26.32±2.38^a^	68.72	C
*A. dracunculus*	IRAN2273C	10.63±1.5^c^	17.19±2.6^a^	83.77	C
	IRAN1018C	25.73±2.6^b^	9.44±3.7^c^	91.65	C
	IRAN2252C	30.17±3.4^a^	41.53±1.2 ^a^	58.15	I

Means followed by the same letter in a column are not significantly different (p≤0.05) compared with Duncan’s Multiple Range Test. T, corrected amount of fungal vegetative and reproductive growth; C, compatibility; I, incompatibility; MGI, mycelial growth inhibition; CI, conidiation inhibition; SE, standard error.

### 3.4 Combined effects of EPF and EOs

**Figure 1:**
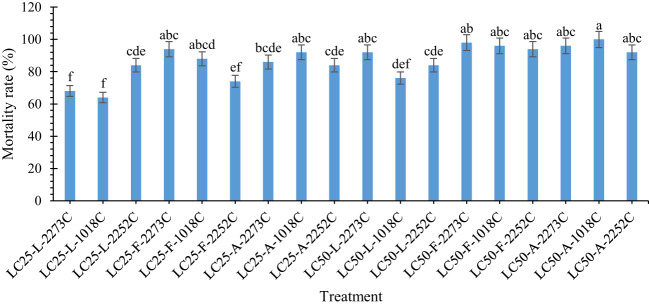
Mortality rate (% ± SE) of *A. obtectus* adults treated with different mixtures of fungal isolates and of plant EOs at LC25 and LC50. In the horizontal axis: (*A.* tarragon, *A. dracunculus*; L: Lavender, *L. angustifolia*; and F: Fennel, *F. vulgare*).

The mortality of *A. obtectus* adults differed significantly among the treatments (F = 17.645; *df* =17, 89; *P* < 0.0001). The highest mortality rate was found following exposure to the mixture of IRAN1018C isolate and LC_50_ of tarragon EO (100% mortality) (**Figure 1**). The lowest insect mortality was found for IRAN1018C isolate and LC_25_ of lavender EO (64% mortality). No significant difference was observed among the mixture of fungal isolates and LC_50_ concentrations of fennel and tarragon EOs. Moreover, the mixtures of IRAN2273C isolate and LC_50_ of lavender EO, IRAN1018C isolate and LC_25_ of tarragon EO, as well as IRAN1018C and IRAN2273C isolates and LC_25_ of fennel EO had the same mortality on *A. obtectus* adults.

According to **Table 4**, the co-application of IRAN1018C isolate with LC_25_ of tarragon and LC_50_ of fennel EOs, and IRAN2273C isolate with LC_50_ of fennel EO showed a synergistic influence on *A. obtectus* mortality. However, the synergic ratio calculated for other combinations was between 0.7-1.8, which shows only additive effects. No antagonistic interaction was observed between combinations (**Table 4**).

**Table 4:** Toxicity of LC_25_ and LC_50_ of essential oils with 10^4^ (spore/ml) of IRAN1018C, IRAN2252C, and IRAN2273C isolates of *M. anisopliae* against adult *A. obtectus* after 24 h.

Essential oil	Fungal isolate	EX		Ob		SR	
		LC_25_	LC_50_	LC_25_	LC_50_	LC_25_	LC_50_
*Laandula angustifolia*	IRAN1018C	47.78	66.78	68	76	0.7	0.87
	IRAN2252C	82	91	84	84	0.85	1.08
	IRAN2273C	86	105	68	92	1.26	1.14
*Foeniculum vulgare*	IRAN1018C	62.78	62.78	88	96	0.71	0.65
	IRAN2252C	86	87	74	94	1.17	0.92
	IRAN2273C	101	101	94	98	1.07	0.62
*Artemisia dracunculus*	IRAN1018C	40.28	78.87	92	100	0.43	0.82
	IRAN2252C	62.5	112	84	92	0.76	1.21
	IRAN2273C	78.5	126	86	96	0.91	1.31

## 4 Discussion

Essential oils have been used traditionally as flavoring and fragrance agents. More recently, their range of use has been extended to human medicine. This subject, together with widespread use in foods and beverages, has described their relative safety *via* empirical practice as well as bioassays in animal models (Isman, 2020). EOs and their constituents are fast-acting neurotoxins in insects and display potentially significant sub-lethal effects in pest insects, including fumigant and contact toxicity, feeding and oviposition deterrence, and repellency (Isman, 2020). Therefore, some companies around the world introduced insecticides based on EOs. For example, in 1998, EOs from rosemary, peppermint, cinnamon, lemongrass, and thyme were used to make commercial essential oil-based insecticides. In addition, some qualified products were produced to control insect pests in urban pest management, greenhouse, horticultural crops, and fruit trees (Isman and Machial, 2006; Isman et al., 2011). However, some problems with essential oil-based insecticides, such as volatility, solubility, and oxidation, significantly affect their activity and application. New formulations, called “Nanoformulation,” help solve the problem. In this case, EOs release in a controlled way through nanocapsule formulations. Therefore, encapsulation of the EOs has a considerable perspective as commercial insecticide products (Martin et al., 2010). In this study, EOs of lavender, fennel, and tarragon exhibited fumigant toxicity against *A. obtectus*. However, insect mortality caused by lavender and fennel EOs was significantly higher than by tarragon. The toxicity of various plant-derived extracts, and EOs against *A. obtectus* has been proved in previous studies. For example, ethanol extract of *L. angustifolia* showed repellent and insecticidal activity against *A. obtectus* adults (Rojht et al., 2012). In another study, EOs from *Ocimum basilicum* L., and *Cymbopogon winterianus* Jowitt affected the development of *A. obtectus*, and the higher concentrations decreased the bean weevil emergence (Rodriguez-González et al., 2019). A similar negative effect on egg-laying and progeny production of *A. obtectus* was observed when exposed to three plant EOs, including eucalyptus (*Eucalyptus camaldulensis* Dehn.), peppermint (*Mentha piperita* L.) and anise (*Pimpinella anisum* L.) (Hategekimana and Erler, 2020). The results of the mentioned studies on *A. obtectus* sensitivity to plant EOs were consistent with the present findings.

Entomopathogenic fungi are the most promising biopesticides due to their current application in controlling many agricultural and public health insect pests. Relevant literature show a variable degree of efficacy for EPF based on their application method, virulence, and insect species (Batta and Kavallieratos, 2018). Several species belonging to the genus *Metarhizium* are among the commonly used biocontrol agents (Litwin et al., 2020). In the current study, although three isolates of *M. anisoploiae*, including IRAN2273C, IRAN2252C, and IRAN1018C, caused 100% mortality in *A. obtectus* adults after six days of treatment, there was a difference among mortality caused by various isolates at the first days during the experiments which may be related to the susceptibility of insects to different isolates of the fungus. On the other hand, the start of the infection process depends on the adhesion of spores on the insect integument and enzyme activity in fungi (Skinner et al., 2014). These two factors may affect the pathogenicity of various isolates. Effective control of insect pests by *M. anisopliae*, consistent with the results of the present study, has been proved in previous studies: Batta (2005) reported more than 50% mortality in seven days for *Rhizopertha dominica* (Fab.) using *M. anisopliae* (Batta, 2005). In another investigation conducted by Vilas Boas et al. (1996), *M. anisopliae* showed more lethality than *B. bassiana* against *Callosobruchus maculatus* (Fabricius) adults (Vilas Boas et al., 1996). These results are consistent with the results of the present study. Rodrigues et al. (1990) reported a reduction in damage made by *Sitophilus zaamais* (Match) and *A. obtectus* using *Beauveria brogniartii* (Sacc.) and *M. anisopliae* as EPF (Rodrigues et al., 1990). Different isolates of *M. anisopliae* var. *acridium* could infect adult insects of pink hibiscus mealybug, *Maconellicoccus hirsutus* Green, within two days after treatment. They caused high mortality in insects (Ujjan and Shahzad, 2008). Using immersion bioassays, various isolates of *M. anisopliae* and *B. bassiana* made adequate control on *C. maculatus* (Cherry et al., 2005). According to Batta and Kavallieratos (2018), no EPF has been registered for commercial use against stored product pests. The possible reasons might be the slower killing effect of EPF compared to chemical insecticides, needing proper formulations with enough water for germination and sporulation of these fungi during the application, and probable defense mechanisms development in target insects. Furthermore, stakeholders in the stored grains resist introducing EPF as biocontrol agents into their facilities because they think these fungi are pathogens or mold. Some solutions like formulating the selected effective strains of EPF as invert emulsions (w/o type), conducting bioassays at a pilot scale or commercial scale under storage conditions using selected formulations, registering the most effective formulations as EPF biopesticides under storage conditions, and using the registered products of EPF commercially at a large scale are recommended (Batta and Kavallieratos, 2018).

Previous studies demonstrated that some EOs might show antimicrobial properties (Hosseinzadeh et al., 2018; Sharifi-Rad et al., 2018). In the current study, the EOs represented a varied degrees of inhibitory action against different isolates of *M. anisopliae*. The highest inhibitory properties on conidiation and mycelial growth belonged to tarragon EOs against IRAN2252C isolate. It is well demonstrated that variation in the fungicidal activity of EOs is related to the differences in their active components, such as phenols, aldehydes, and ketones (Oussalah et al., 2007). In a study by Hosseinzadeh et al. (2018), EOs from parsley (*Petroselinum sativum* Mill.), angelica (*Heracleum persicum* Desf. Ex Fisch.), and safflower (*Satureja sahendica* Bornm.) inhibited mycelial growth of *B. bassiana* isolate Is-75. There was a direct relationship between fungal growth inhibition and conidiation which agreed with the results of this study. Adversely, in some studies, fungal growth did not alter by EOs. For example, according to Borgio et al. (2008) various extracts from leaves, roots, stems, and seeds of *Ocimum sanctum* did not affect the conidial production of *M. anisopliae* (Borgio et al., 2008). In another study investigating the compatibility of some EPF and the neonicotinoid insecticides, acetamiprid increased the vegetative growth of *Paecilomyces* sp. (Neves et al., 2001). It might be due to physiological resistance mechanisms in fungi that metabolize the insecticides and utilize the released compounds as a secondary nutrient. Alternatively, fungi may expand their reproductive activities in a toxic media, which can result in more conidia production (Neves et al., 2001). Our results showed that tarragon EO was incompatible with IRAN2252C isolate. However, lavender and fennel EOs did not have an entirely negative effect on the fungal isolates, even if reduced mycelial growth and conidiation were detected.

To increase the effectiveness of EOs and EPF, *M. anisopliae* var. *acridum* and *B. bassiana* were applied simultaneously with the EOs of parsley, cumin, and onion against *Schistocerca gregaria* (Forskal) and *Euprepocnemis plorans* (Charpentier). According to the results, combining parsley and cumin EOs with *M. anisopliae* was the most effective treatment (Mohamed, 2009). The isolated and simultaneous effects of *Acalypha alnifolia* Klein ex Willd. leaf extract and *M. anisopliae* against the malaria mosquito *Anopheles stephensi* Liston. indicated promising larvicidal and pupicidal properties (Murugan et al., 2012). In the study of separate and simultaneous effects of *M. piperita* and *Mentha pulegium* L. EOs and the pathogenic fungus *Lecanicilium muscarium* against *Aphis gossypii* Glover, the combination of EOs and EPF had the potential to manage the pest (Ebadollahi et al., 2017). In all of the literature mentioned above, the combined effect of EPF and EOs is additive or synergist, which agrees with the results of the current study. On the contrary, interactions between sublethal concentrations of *P. sativum*, *S. sahendica*, and *H. persicum* EOs and IS-1 and IS-75 isolates of *Beauveria bassiana* against *C. maculatus* revealed that except for the LC_25_ combination of agents with synergistic effect, other sublethal combinations showed additive or antagonistic effects on adults’ mortality (Hosseinzadeh et al., 2018).

## 5 Conclusion

The application of entomopathogenic fungi and plant essential oils as natural control agents should result in fewer harmful side effects compared to synthetic chemical insecticides. According to the present findings, the combination of fungal isolates and plant EOs seems effective for insect pest control. The control of bean weevil, *A. obtectus*, benefited from the combining effects of EPF and EOs; however, their performance depended on the combination. Therefore, the interactive effect of EOs on the mycelial development and conidiation of fungal isolates should be examined before application. The presented results showed additive or synergy properties of integrated application of *A. dracunculus*, *F. vulgare*, and *L. angustifolia* EOs and entomopathogenic fungus *M. anisoplia* for managing *A. obtectus*. More studies are still needed to evaluate the separate and combined effects of these agents in warehouses.

## Data availability statement

The raw data supporting the conclusions of this article will be made available by the authors, without undue reservation.

## Author contributions

NZ-S and MHGP conceived and designed the research. FL performed the experiments. NZ-S and MHGP wrote the manuscript and AE revised it. All authors contributed to the article and approved the submitted version.

## References

[B1] Abbott W. S. (1925). A method of computing the effectiveness of an insecticide. J. Econ. Entomol. 18(2), doi: 265-267. 3333059

[B2] Akbar W. Lord J. C. Nechols J. R. Loughin T. M. (2005). Efficacy of *Beauveria bassiana* for red flour beetle when applied with plant essential oils or in mineral oil and organosilicone carriers. J. Econ. Entomol. 98, 683–688. doi: 10.1603/0022-0493-98.3.683 16022293

[B3] Batta Y. A. (2005). Control of the lesser grain borer (*Rhyzopertha dominica* (F.), coleoptera: Bostrichidae) by treatments with residual formulations of *Metarhizium anisopliae* (Metschnikoff) sorokin (Deuteromycotina: Hyphomycetes). J. Stored Prod. Res. 41, 221–229. doi: 10.1016/j.jspr.2004.03.007

[B4] Batta Y. A. Kavallieratos N. G. (2018). The use of entomopathogenic fungi for the control of stored-grain insects. Int. J. Pest Manage. 64, 77–87. doi: 10.1080/09670874.2017.1329565

[B5] Borgio J. F. Bency B. J. Sharma N. (2008). Compatibility of *Metarhizium anisopliae* ( metsch .) sorok . with *Ocimum sanctum* Linn . ( tulsi ) ( lamiaceae ) extracts. Ethnobot. Leafl. 12 12, 698–704.

[B6] Cherry A. J. Abalo P. Hell K. (2005). A laboratory assessment of the potential of different strains of the entomopathogenic fungi *Beauveria bassiana* (Balsamo) vuillemin and *Metarhizium anisopliae* (Metschnikoff) to control *Callosobruchus maculatus* (F.)(Coleoptera: Bruchidae) in stored cowpea. J. Stored Prod. Res. 41, 295–309. doi: 10.1016/j.jspr.2004.04.002

[B7] Daglish G. J. Nayak M. K. Arthur F. H. Athanassiou C. G. (2018). “Insect pest management in stored grain,” in Recent advances in stored product protection (Berlin Heidelberg: Springer), 45–63.

[B8] Ebadollahi A. Davari M. Razmjou J. Naseri B. (2017). Separate and combined effects of *Mentha piperata* and *Mentha pulegium* essential oils and a pathogenic fungus *Lecanicillium muscarium* against *Aphis gossypii* (Hemiptera: Aphididae). J. Econ. Entomol. 110, 1025–1030. doi: 10.1093/jee/tox065 28334238

[B9] Ebadollahi A. Jalali Sendi J. (2015). A review on recent research results on bio-effects of plant essential oils against major coleopteran insect pests. Toxin Rev. 34, 76–91. doi: 10.3109/15569543.2015.1023956

[B10] Ebadollahi A. Ziaee M. Palla F. (2020). Essential oils extracted from different species of the lamiaceae plant family as prospective bioagents against several detrimental pests. Molecules 25, 1556. doi: 10.3390/molecules25071556 32231104 PMC7180760

[B11] Eckard S. Bacher S. Enkerli J. Grabenweger G. (2017). A simple in vitro method to study interactions between soil insects, entomopathogenic fungi, and plant extracts. Entomol. Exp. Appl. 163, 315–327. doi: 10.1111/eea.12578

[B12] Farzaneh M. Kiani H. Sharifi R. Reisi M. Hadian J. (2015). Chemical composition and antifungal effects of three species of Satureja (S. hortensis, S. spicigera, and S. khuzistanica) essential oils on the main pathogens of strawberry fruit. Postharvest Biol. Technol. 109, 145–151. doi: 10.1016/j.postharvbio.2015.06.014

[B13] Ghahari H. Borowiec L. (2017). A checklist of seed-beetles (Coleoptera: Chrysomelidae: Bruchinae) from Iran. Zootaxa 4268, 215–237. doi: 10.11646/zootaxa.4268.2.3 28610372

[B14] Hategekimana A. Erler F. (2020). Fecundity and fertility inhibition effects of some plant essential oils and their major components against *Acanthoscelides obtectus* say (Coleoptera: Bruchidae). J. Plant Dis. Prot. 127, 615–623. doi: 10.1007/s41348-020-00311-3

[B15] Hosseinzadeh R. Mehrvar A. Eivazian K. N. (2018). Compatibility of some plant essential oils in combination with the entomopathogenic fungus, *Beauveria bassiana* against *Callosobruchus maculatus* (Col.: Bruchidae). Plant Pests Res. 8, 1–14. doi: 10.22124/IPRJ.2018.2834

[B16] Isman M. B. (2020). Commercial development of plant essential oils and their constituents as active ingredients in bioinsecticides. Phytochem. Rev. 19, 235–241. doi: 10.1007/s11101-019-09653-9

[B17] Isman M. B. Grieneisen M. L. (2014). Botanical insecticide research: many publications, limited useful data. Trends Plant Sci. 19, 140–145. doi: 10.1016/j.tplants.2013.11.005 24332226

[B18] Isman M. B. Machial C. M. (2006). Pesticides based on plant essential oils: from traditional practice to commercialization. Adv phytomedicine. 3, 29–44. doi: 10.1016/S1572-557X(06)03002-9

[B19] Isman M. B. Miresmailli S. Machial C. (2011). Commercial opportunities for pesticides based on plant essential oils in agriculture, industry and consumer products. Phytochem. rev. 10(2), 197–204. doi: 10.1007/s11101-010-9170-4

[B20] Kovendan K. Murugan K. Vincent S. (2012). Evaluation of larvicidal activity of *Acalypha alnifolia* Klein ex willd. (Euphorbiaceae) leaf extract against the malarial vector, *Anopheles stephensi*, dengue vector, *Aedes aegypti* and bancroftian filariasis vector, *Culex quinquefasciatus* (Diptera: Culicid. Parasitol. Res. 110, 571–581. doi: 10.1007/s00436-011-2525-y 21748350

[B21] Litwin A. Nowak M. Różalska S. (2020). Entomopathogenic fungi: unconventional applications. Rev. Environ. Sci. Biotechnol. 19, 23–42. doi: 10.1007/S11157-020-09525-1

[B22] Martin Á. Varona S. Navarrete A. Cocero M. J. (2010). Encapsulation and co-precipitation processes with supercritical fluids: applications with essential oils. Open Chem. Eng. J. 4, 31–41. doi: 10.2174/1874123101004010031

[B23] Mohamed G. A. (2009). Increasing the efficacy of *Metarhizium anisopliae* var. acridum (Metchnikoff) soroken and *Beauveria bassiana* (Bals.) vuill. using certain essential oils against desert locust and grasshoppers. Egypt. J. Biol. Pest Control 19, 67–72.

[B24] Murugan K. Kovendan K. Vincent S. Barnard D. R. (2012). Biolarvicidal and pupicidal activity of acalypha alnifolia Klein ex Willd.(Family: Euphorbiaceae) leaf extract and microbial insecticide, metarhizium anisopliae (Metsch.) against malaria fever mosquito, anopheles stephensi Liston.(Diptera: Culicidae). Parasitol. Res. 110, 2263–2270. doi: 10.1007/s00436-011-2758-9 22200954

[B25] Murugan K. Madhiyazhagan P. Thiyagarajan N. Radha R. Murugan K. Wei H.. (2014) Insecticidal activity of essential oils and entomopathogenic fungi against cowpea bruchid, callosobruchus maculatus (f.) (Insecta: Coleoptera: bruchidae). Available at: https://www.researchgate.net/publication/269990140.

[B26] Napoleão T. H. Agra-Neto A. C. Belmonte B. R. Pontual E. V. Paiva P. M. G. (2015). “Biology, ecology and strategies for control of stored-grain beetles: a review,” in Beetles biodiversity (New York: Ecol. role Environ. Nov. Sci. Publ. Inc.), 105–122. doi: 10.1007/s00436-011-2758-9

[B27] Nayak M. K. Daglish G. J. (2018). “Importance of stored product insects,” In Recent advances in stored product protection (Springer, Berlin, Heidelberg: Springer), 1–17.

[B28] Neves P. M. O. J. Hirose E. Tchujo P. T. Moino A. (2001). Compatibility of entomopathogenic fungi with neonicotinoid insecticides. Neotrop. Entomol. 30, 263–268. doi: 10.1590/s1519-566x2001000200009

[B29] Nyamador W. S. Ketoh G. K. Amévoin K. Nuto Y. Koumaglo H. K. Glitho I. A. (2010). Variation in the susceptibility of two *Callosobruchus* species to essential oils. J. Stored Prod. Res. 46, 48–51. doi: 10.1016/j.jspr.2009.09.002

[B30] Oussalah M. Caillet S. Saucier L. Lacroix M. (2007). Inhibitory effects of selected plant essential oils on the growth of four pathogenic bacteria: E. coli O157: H7, *Salmonella typhimurium*, *Staphylococcus aureus* and *Listeria monocytogenes* . Food Control 18, 414–420. doi: 10.1016/j.foodcont.2005.11.009

[B31] Panahi O. Ghane Jahromi M. Loni A. (2014). Compatibility and study effect of some insecticides and essential oils with entomopathogenic fungus, in laboratory condition. IAU Entomol. Res. J. 6(3), 203–213.

[B32] Parsons D. M. J. Credland P. F. (2003). Determinants of oviposition in *Acanthoscelides obtectus*: a nonconformist bruchid. Physiol. Entomol. 28, 221–231. doi: 10.1046/j.1365-3032.2003.00336.x

[B33] Reddy G. V. P. Zhao Z. Humber R. A. (2014). Laboratory and field efficacy of entomopathogenic fungi for the management of the sweetpotato weevil, *Cylas formicarius* (Coleoptera: Brentidae). J. Invertebr. Pathol. 122, 10–15. doi: 10.1016/j.jip.2014.07.009 25111763

[B34] Rodrigues C. Pratissoli D. (1990). Pathogenicity of *Beauveria brongniartii* (Sacc.) petch and *Metarhizium anisopliae* (Mots.) sorok and their effect on the corn weevil and the bean beetle. An. da Soc Entomol. do Brasil 19 (2), 301–306. doi: 10.37486/0301-8059.v19i2.659

[B35] Rodriguez-González Á. Álvarez-Garcia S. González-López Ó. Da Silva F. Casquero P. A. (2019). Insecticidal properties of *Ocimum basilicum* and *Cymbopogon winterianus* against *Acanthoscelides obtectus*, insect pest of the common bean (Phaseolus vulgaris, l.). Insects 10, 151. doi: 10.3390/insects10050151 31130631 PMC6572361

[B36] Rojht H. Košir I. J. Trdan S. (2012). Chemical analysis of three herbal extracts and observation of their activity against adults of *Acanthoscelides obtectus* and *Leptinotarsa decemlineata* using a video tracking system. J. Plant Dis. Prot. 119, 59–67. doi: 10.1007/BF03356421

[B37] Schalk J. M. Rassoulian G. (1973). Callosobruchus maculatus: Observations of attack on cowpeas in Iran. J. Econ. Entomol. 66, 579–580. doi: 10.1093/jee/66.2.579

[B38] Sharifi-Rad M. Varoni E. M. Iriti M. Martorell M. Setzer W. N. del Mar Contreras M.. (2018). Carvacrol and human health: A comprehensive review. Phyther. Res. 32, 1675–1687. doi: 10.1002/PTR.6103 29744941

[B39] Skinner M. Parker B. L. Kim J. S. (2014). “Role of entomopathogenic fungi in integrated pest management,” in Integrated pest management: Current concepts and ecological perspective (Cambridge, Massachusetts, United States: Academic Press), 169–191. doi: 10.1016/B978-0-12-398529-3.00011-7

[B40] Tharanathan R. N. Mahadevamma S. (2003). Grain legumesa boon to human nutrition. Trends Food Sci. Technol. 14, 507–518. doi: 10.1016/j.tifs.2003.07.002

[B41] Tripathi A. K. (2018). “Pests of stored grains,” in Pests and their management (Berlin Heidelberg: Springer), 311–359.

[B42] Ujjan A. A. Shahzad S. (2008). Pathogenicity of *Metarhizium anisopliae* var. acridum strains on pink hibiscus mealy bug (*Maconellicoccus hirsutus*) affecting cotton crop. Pakistan J. Bot. 39, 967–973.

[B43] Vilas Boas A. M. Oliveira J. V. Campos A. L. Andrade R. M. Silva R. L. X. (1996). Pathogenicity of wild strains and mutants of metarhizium anisopliae and *Beauveria bassiana* to *Callosobruchus maculatus* (Fab. 1792)(Coleoptera, bruchidae). Arq. Biol. e Tecnol. 39, 99–104.

